# Mother–child neural synchronization is time linked to mother–child positive affective state matching

**DOI:** 10.1093/scan/nsad001

**Published:** 2023-01-27

**Authors:** Judith K Morgan, Hendrik Santosa, Kaetlyn K Conner, Rachel M Fridley, Erika E Forbes, Satish Iyengar, Heather M Joseph, Theodore J Huppert

**Affiliations:** Department of Psychiatry, University of Pittsburgh, Pittsburgh, PA 15260, USA; Department of Psychology, University of Pittsburgh, Pittsburgh, PA 15260, USA; Department of Radiology, University of Pittsburgh, Pittsburgh, PA 15260, USA; University of Pittsburgh Medical Center, Pittsburgh, PA 15260, USA; University of Pittsburgh Medical Center, Pittsburgh, PA 15260, USA; Department of Psychiatry, University of Pittsburgh, Pittsburgh, PA 15260, USA; Department of Psychology, University of Pittsburgh, Pittsburgh, PA 15260, USA; Department of Statistics, University of Pittsburgh, Pittsburgh, PA 15260, USA; Department of Psychiatry, University of Pittsburgh, Pittsburgh, PA 15260, USA; Department of Electrical and Computer Engineering, University of Pittsburgh, Pittsburgh, PA 15260, USA

**Keywords:** synchrony, positive Affect, prefrontal Cortex

## Abstract

In the first years of life, in which self-regulation occurs via external means, mother–child synchronization of positive affect (PA) facilitates regulation of child homeostatic systems. Mother–child affective synchrony may contribute to mother–child synchronization of neural systems, but limited research has explored this possibility. Participants were 41 healthy mother–child dyads (56% girls; *M*age = 24.76 months; *s.d.* = 8.77 months, *Range *= 10–42 months). Mothers’ and children’s brain activities were assessed simultaneously using near-infrared spectroscopy while engaging in dyadic play. Mother and child PA during play were coded separately to characterize periods in which mothers and children (i) matched on high PA, (ii) matched on low/no PA or (iii) showed a mismatch in PA. Models evaluated moment-to-moment correlations between affective matching and neural synchrony in mother–child dyads. Greater positive affective synchrony, in which mother and child showed *similarly high* levels of PA but not similarly low levels of PA, was related to greater synchrony in medial and lateral frontal and temporoparietal regions. Age moderated associations between mother and child neural activities but only during moments of high PA state matching. Positive, synchronous mother–child interactions may foster greater neural responding in affective and social regions important for self-regulation and interpersonal bonds.

Affective state matching, in which mothers and children imitate and elaborate on each other’s emotional expressions, is a hallmark characteristic of early mother–child interactions (Bell, 2020). In the context of positive emotions, affective state matching involves shared smiling, mutual eye gaze, back-and-forth vocalizations and joint play (Feldman, 2007, 2012). This process of responding to one another’s positive affective bids by imitation and elaboration serves to maintain and prolong the positive interaction between mother and child (Leclère *et al.*, 2014), which may contribute to a flexible, supportive mother–child relationship in the long term.

Over time, positive affective state matching also appears to promote child emotional self-regulation through co-regulation, the process in which mothers and children dynamically alter their emotions, behavior and physiology in the context of one another (Feldman, 2015; Noe *et al*., 2015). Co-regulation appears to occur by virtue of dyadic synchronization of physiology. Specifically, greater dyadic matching of positive emotions was associated with stronger synchronization of heart rhythms during that interaction in a study of mother–infant dyads (Feldman *et al*., 2011b). In a separate study, mother–child dyads with more reciprocal and responsive interactions (e.g. greater positive emotion and joint play) showed more concordant release of oxytocin during the interaction (Feldman *et al*., 2011a). These findings suggest that affective state matching may reinforce synchronization of physiologic systems in mother–child dyads, which over time may support the child’s self-regulation of these physiologic systems accordingly (Feldman *et al.*, 1999; Feldman, 2015; Wass *et al*., 2019; Abney *et al*., 2021).

Although the link between affective state matching and synchronization of physiologic systems has been established, less is known about whether affective state matching may also be associated with synchronization of mother–child neural systems during the sensitive first years of life. Given that mother–child interactions characterized by shared emotion expression and responsive behavior require activity in neural regions involved in these emotional and social processes such as prefrontal cortex (PFC) and temporoparietal junction (TPJ), synchronization of positive affective behavior likely also involves synchronization of these neural regions as well. Prior research studies have demonstrated that caregivers and children show more synchronous brain activity in the context of shared eye gaze, gentle touch and simultaneous vocalization (Wass *et al*., 2020; Nguyen *et al*., 2021a). Along those lines, positive affective state matching likely also elicits mother–child synchronization of brain activity in neural regions involved in positive emotion expression given that positive, harmonious interactions are characterized by moments of shared smiling, mutual eye gaze, gentle touch and greater vocalizations (Feldman, 2007).

A set of prefrontal regions contributing to affective and social circuitry could be particularly important to sustaining mother–child synchronous behavior. The anterior medial PFC is a neural region that has been implicated in emotion expression, affectionate touch and social cognition (Spielberg *et al*., 2008; Etkin *et al*., 2011; Grossmann, 2013). The lateral PFC is a regulatory region that has been demonstrated to promote both down- and up-regulation of emotion (Kim and Hamann, 2007). Both the anterior medial and lateral PFCs are developing rapidly in the first years of life (Grossmann, 2013; Hodel, 2018). Indeed, recent reviews of developmental changes in prefrontal activity in the first years of life (see Grossmann, 2013; Hodel, 2018) have revealed that the medial PFC is actively recruited in response to facial, but not object stimuli, as early as the first months of life and that the lateral PFC responds to auditory stimuli in the first months of life (Nakano *et al*., 2009; Tzourio-Mazoyer *et al*., 2002). Importantly, prior meta-analytic reviews outline the rapid changes in PFC volume and activity that occur in the first 2 years of life and the ways in which early experiences influence these early changes (Hodel, 2018).

The TPJ is a social region thought to be involved in mentalizing or the ability to understand the thoughts and feelings of others (Frith and Frith, 2006; Decety and Lamm, 2007). Similar to the PFC, function within the TPJ is developing rapidly in the first years of life to facilitate children’s ability to make sense of their social world. For example, one study using near-infrared spectroscopy (NIRS) during mother–child dyadic interactions demonstrated that the TPJ is already specialized by 12–14 months of age to process social signals (Hakuno *et al*., 2018). Other work has shown significant increases in neural activity in the TPJ in response to social stimuli across the first 24 months of life (Lloyd-Fox *et al*., 2017).

Mother–child affective state matching that promotes mother–child synchronization of neural activity within prefrontal and temporoparietal regions could support development of neural systems and pathways in children over time. Indeed, newly emerging work has demonstrated that maternal affectionate touch during mother–infant interactions is related to stronger neural synchrony in the lateral and medial PFCs in a sample of mothers and 4- to 6-month-old infants (Nguyen *et al*., 2021a). Development of function in the prefrontal and temporoparietal regions can foster self-regulated emotion expression and prosocial behavior, important transdiagnostic protective factors (Keenan, 1994). One reason for the limited understanding of mother–child neural synchronization is due to methodological limitations with traditional neuroimaging paradigms (e.g. Functional Magnetic Resonance Imaging), which prohibit observation of coordinated neural function during naturalistic, free-moving behavior. NIRS is a nonrestrictive optical imaging method that is less sensitive to movement relative to other imaging modalities, making it advantageous for the assessment of neural activity in a young child population and in naturalistic contexts (e.g. dyadic play interactions) (Aslin and Mehler, 2005; Lloyd-Fox *et al*., 2010). In particular, the use of NIRS hyperscanning, in which two or more people can be assessed simultaneously using the same system, is a valuable advance that allows for the assessment of mother–child neural synchronization during *in vivo* mother–child play. This innovative strategy allows for the assessment of *moment-to-moment* changes in mother–child brain activity *while* they engage in naturalistic, interactive play.

Newly emerging work using NIRS hyperscanning with young infants (<12 months) or with preschool age children (4- to 5-year-olds) has demonstrated that the degree of mother–child neural synchrony is predicted by a number of parental (e.g. maternal stress or attachment anxiety) *or* child characteristics (e.g. child irritability) (Azhari *et al*., 2019, 2020; Quiñones-Camacho *et al*., 2020). One study evaluated how mother–child interaction quality, measured globally, was related to mother–child neural synchrony and found that higher levels of globally coded mother–child cooperation vs. individual problem-solving were associated with increased neural synchrony in the bilateral PFC and temporoparietal regions compared to globally coded mother–child competition (Nguyen *et al*., 2020). However, few studies have evaluated how moment-to-moment changes in affective state matching within the dyad are related to mother–child neural synchrony. (Nguyen *et al*. 2021a) found that microanalytic-coded affective synchrony and neural synchrony were unrelated in a sample of 4- to 6-month-olds and their mothers. This is important as evaluation of momentary behavior (vs. global behavior averaged over time) within the dyad can better elucidate transactional processes that shape brain functions in children.

Further, despite a growing body of work examining neural synchrony in young infants <12 months old (Nguyen *et al*., 2021a), in older 4- to 5-year-old preschoolers (Quiñones-Camacho *et al*., 2020; Nguyen *et al*., 2021b) or in school-age children (Reindl *et al*., 2018), few studies have evaluated neural synchrony in dyads with very young children between 1 and 3 years of age (but see Azhari *et al*., 2020). The period between 1 and 3 years of age is characterized by remarkable changes in child self-regulatory abilities that are occurring in the context of the caregiving environment (Kopp, 1982), making it a clinically important period of investigation. Understanding developmental brain changes that occur across this period of rapid development from ages 1 to 3 is important because it can elucidate how changes in brain response in the context of the caregiving environment may support healthy emotional development during this sensitive developmental period (Hodel, 2018). Altogether, evaluation of mother–child neural synchrony in the context of momentary changes in mother–child affective state matching during the first 3 years of life would elucidate how mother–child interactions shape child emotional and brain development via synchronization of mother–child brain activities (Wass *et al*., 2020).

Because prior work has established the unique and important role of mother–child synchronization of positive affective behavior (rather than negative affective behavior) on child regulatory development (Lobo and Lunkenheimer, 2020), we evaluated whether greater positive affective state matching between mothers and their very young children (1- to 3-year-olds) would be associated with stronger associations between emotion expression, emotion regulation and mentalizing regions, specifically the anterior medial, lateral prefrontal and TPJ. We also explored how child age may moderate the association between positive affective state matching and mother–child neural synchrony. We utilized an approach that allowed for moment-to-moment assessment of mother and child affect and mother and child brain activities. We hypothesized that there would be greater synchronization of neural activity in the anterior medial, lateral prefrontal and temporoparietal regions in moments in which mothers and children matched on high levels of positive affect (PA), relative to when they matched on low PA or had a mismatch in PA.

## Methods

Participants were 41 1- to 3-year-old typically developing children (56% female; *M*age = 24.76 months, s.d. = 8.77, *Range *= 10–42 months) and their birth mothers (*M*age = 32.93 years, s.d. = 3.42, *Range *= 26–39 years). Of these 41 children, 61% were identified as White, 15% as Black/African American, 12% as Asian and 12% as multiracial; 7% identified as Latinx. Of the 41 mothers, 66% were identified as White, 17% as Black/African American, 12% as Asian and 5% as multiracial; 2% identified as Latinx. The majority (90%) of mothers reported being married (*n *= 34) or living with a partner (*n *= 3) and 10% reported being single (*n *= 3) or separated (*n *= 1). The majority of mothers (83%) also reported having some college-level education. All mothers were free of current and past psychiatric illness, and children were typically developing and free of neurological problems. An additional six dyads were invited to complete the study protocol; however, due to child refusal to wear the cap (*n *= 3), poor cap placement (*n *= 2) or technical issues (*n *= 1), they were not included in the study. The six dyads without NIRS data did not significantly differ from the 41 dyads with NIRS data in terms of mother’s age (*t *= 0.46, *p *= 0.65), mother education (*χ*^2^ = 1.65, *p *= 0.65) or mother relationship status (*χ*^2^ = 4.19, *p *=0.38) or in terms of child age (*t *= 0.45, *p *= 0.51, *M*age = 20.83 months), race (*χ*^2^ = 2.40, *p *= 0.49), ethnicity (*χ*^2^ = 0.47, *p* = 0.99) or sex (*χ*^2^ = 1.61, *p *= 0.38).

### Procedure

**Fig. 1.**
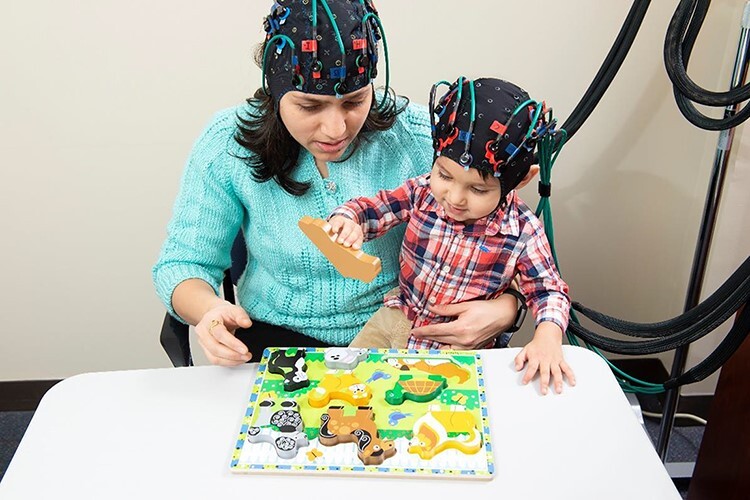
Image of mother–child synchrony task.

Mother–child dyads participated in a structured play task in which they engaged with a developmentally appropriate toy for 3 min. During this task, mother and child neural activity were assessed simultaneously using a TechEn CW6 NIRS system (i.e. hyperscanning, see Figure 1). This task was chosen because engaging with a toy together is an ecologically valid task that mothers and children of this age typically enjoy. Furthermore, engagement with a toy facilitated successful child compliance with the NIRS assessment. The study was approved by the University of Pittsburgh Human Research Protections Office, and mothers provided informed consent prior to participation.

### Measures

#### Mother–child play

Each mother was seated at a table with her young child and asked to play with an age-appropriate toy with her child, who was seated in her lap, for 3 min. Dyads were either given a set of stacking rings (for 1-year-old children) or a wooden block puzzle (for 2- or 3-year-old children). Each mother was told to play and interact with her young child as she normally would at home using this toy. Both mother and child brain activity were assessed via NIRS during the 3 min toy play interaction, as it increased child compliance with NIRS assessment and allowed for the assessment of brain activity during a naturalistic interaction with minimal movement.

#### Affective coding

Independent observers naïve to study hypotheses coded mothers and children on PA and negative affect (NA) in 5 s epochs or ‘moments’ across the entire 3 min task (i.e. 36 moments). Five-second epochs were used to ensure that the behavioral coding window was sensitive to within-person changes in affective behavior while still capturing neural activity occurring in response to the observed behavior due to the sluggish nature of the hemodynamic response (i.e. 2–5 s; Hong *et al*., 2015). Mothers and children were scored separately by trained coders on the same moment. Based on the AFFEX coding system (Izard *et al*., 1983) and similar to other research (Morgan *et al*., 2020), PA was coded dichotomously as the presence of vocal, facial and bodily expressions of happy/joyful affect (0 = no/low PA, 1 = high PA). In particular, vocal expressions of PA included exclamations of joy (‘wow!’, ‘yay!’) and lilting voices, facial expressions of PA included smiling and brightening of eyes and bodily expressions of PA included clapping hands. NA was coded dichotomously as the presence of vocal, facial and bodily expressions of sadness, anger/frustration or fear (0 = no/low NA, 1 = high NA). In particular, vocal expressions of NA included crying or screaming, facial expressions of NA included pouting lip or frowning and bodily expressions of NA included tensing body. Coders were trained to 80% reliability and periodically retrained to prevent coder drift. Eight videos (19%) were then double coded with the primary coder who had graduate-level training in the AFFEX coding system (Izard *et al*., 1983), and reliability was high for both mother and child PA (intraclass correlation coefficient (ICC) = 0.93 for mother and 0.95 for child) and mother and child NA (ICC = 0.90 for mother and 0.95 for child). Due to the relatively low base rate of NA (1.4% of task for mothers, 7.4% of task for children) for this inherently pleasant task (i.e. joint toy play) and our interest in neural synchronization in the context of positive, harmonious interactions, we focused on positive affective state matching for analyses.

#### Characterization of affective matching conditions

Depending on mother and child PA coding, each moment was categorized into one of four groups: (i) matching on high PA, (ii) matching on low PA, (iii) mismatched PA in mother displayed high PA and child displayed low PA or (iv) mismatched PA in which mother displayed low PA and child displayed high PA during that moment. Seven children had an abbreviated mother–child toy play task due to child fussiness (*M*time = 2:16, s.d. = 31 s), resulting in 1411 coded moments (out of possible 1476).

### Functional Near Infrared Spectroscopy (fNIRS) data collection

Optical imaging was performed using a continuous-wave CW6 NIRS system (TechEn, Milford, MA) at a sampling rate of 20 Hz. The data were measured simultaneously at two wavelengths, 690  and 830 nm. Light intensity was automatically adjusted by the system to provide optimal gain. Fiber optics were split between two caps worn by both members of the dyad, which allowed for simultaneous measurement of mother and child brain activities (i.e. hyperscanning). A total of eight channels were measured from four sources and seven detectors for the mother. A total of 12 channels were measured from six sources and nine detectors for the child. The child probe featured more channels due to the larger project’s focus on longitudinal changes in child neural response to reward-related stimuli. The distance between each source and detector was 3 cm. Sensors were mounted on a neoprene cap sized based on head circumference. The probe extended over the inferior frontal gyrus, including the anterior medial PFC (Brodmann Area 10) to the parietal regions for both mother and child (Whiteman *et al*., 2017). For each participant, the NIRS cap was positioned according to the international 10–20 coordinate system at the center of the lower edge of the probe (detector 5 for child, detector 13 for mother) aligned with FpZ.

### Preprocessing and analytic plan

The NIRS data were analyzed in Matlab^TM^ (MathWorks, Natick, MA, 2021) as part of an open-source AnalyzIR toolbox (Santosa *et al.*, 2018). Raw NIRS signals were first resampled to 4 Hz and converted to changes in optical density. Then, the measured intensity data of the two wavelengths were converted to relative oxy- and deoxy-hemoglobin concentration changes using the modified Beer–Lambert law (Cope *et al*., 1998). An instantaneous correlation time course was computed from all 12 (child) × 8 (mother) channels (48 unique time courses for both oxy- and deoxy-hemoglobin). The time course of each mother–child channel pair was then used as a dependent variable in an autoregressive model to correct for noise in the data (Barker *et al*., 2013). This previously validated model has been demonstrated to show better sensitivity–specificity characteristics and statistically addresses both increased false discovery rates introduced by serially correlated noise due to physiology in NIRS and outliers related to motion (Huppert, 2016; Santosa *et al.*, 2018). A design matrix that encodes the mother PA, child PA and their four (2-by-2) joint states was included as a regressor.

Next, for second-level analyses, we used a group-level statistical fixed effects model to evaluate moment-to-moment associations between the mother–child NIRS channel time courses and the mother–child affective matching conditions (see *Technical Supplement* for more details). To account for variability in groupings (i.e. matching on low PA was more common than other groupings), we used permutation tests to perform a nonparametric statistical inference of the second-level model in which we estimated a null distribution (i.e. creating null dyads in which a participating mother was matched with an unrelated child) and ran 20 000 iterations for each grouping at each region of interest channel to compare true dyads with null dyads and to ensure that our findings were specific to true dyads (Fishburn *et al*., 2018). Follow-up analyses evaluated age-related differences in mother–child synchrony by including child age as a fixed effect in models. As previously done in other neuroimaging studies (Carter *et al*., 2016), only findings significant at *p *< 0.005 are presented to reduce Type 1 error. We examined whether findings survived corrections for multiple comparisons using a false discovery rate (*q*) < 0.05 (Benjamini–Hochberg). Although none of our findings survived these corrections, we note the corrected *p* (*q*) for each finding.

## Results

### Descriptive statistics

On average, children displayed PA during 15% of the task (*M *= 5.57 epochs out of 36) and mothers displayed PA during 22% of the task (*M *= 7.98 epochs out of 36). Mothers and children matched on low PA 68.6% of the time (968 epochs); matched on high PA 6.4% of the time (91 epochs); had mismatched PA in which the mother displayed high PA, but child displayed low PA 15.7% of epochs (221 epochs), or had mismatched PA in which the child displayed high PA, but mother displayed low PA 9.3% of epochs (131 epochs).

All dyads matched on low PA at least once during the task (*Mepochs *= 23.61, s.d. = 8.40) and 20 matched on high PA at least once during the task (*Mepochs *= 2.22, s.d. = 3.66). Furthermore, 28 had a mismatch in which children showed high PA and mothers showed low PA (*Mepochs *= 3.20, s.d. = 4.59) at least once during the task, and 37 had a mismatch in which children showed low PA and mothers showed high PA (*Mepochs *= 5.39, s.d. = 5.40) at least once during the task. Thus, analyses were conducted on the sample of epochs (*n *= 1411) across participants (i.e. on the epoch level vs. the dyad level) (see Table 1). There were no differences in dyads with and without full grouping data (i.e. all match/mismatch types) in terms of child age (*t *= −1.17, *p *= 0.25) or sex (*χ*^2^ = 0.49, *p *= 0.54).

**Table 1.** Number of epochs and dyads by grouping

Grouping	Number of epochs in analyses	Number of dyads in analyses
Matched on high PA	91	20
Matched on low PA	968	41
Mismatch (child high PA, mother low PA)	131	28
Mismatch (child low PA, mother high PA)	221	37

### Linking affective matching with mother–child neural synchrony

#### Mother–child matching of high PA

**Fig. 2.**
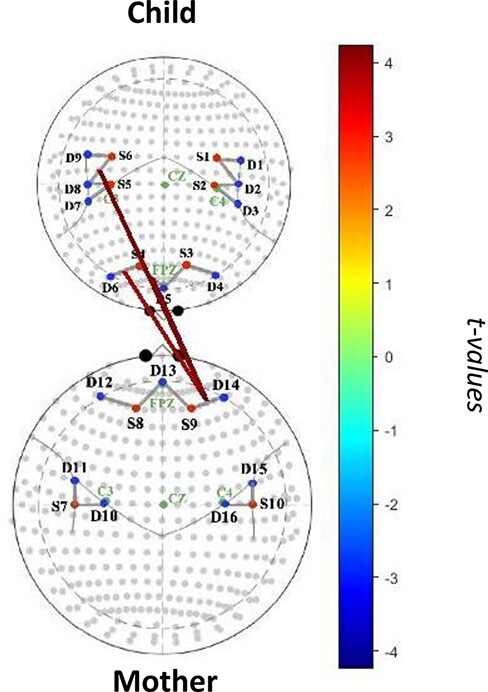
Mother–child neural synchrony during high-intensity positive affective state matching.

Greater positive affective synchrony was associated with greater synchrony in medial frontal, lateral frontal and temporoparietal regions (see Table 2, Figure 2). More specifically, in moments in which mother and child showed similarly high levels of PA, there was a significant association between mother and child neural activities in the right lateral PFC (*β *= 0.32, *t *= 3.81, *p *= 0.0048, *q *= 0.47). There was also a significant positive association between neural activity in the mother right lateral PFC and activity in child anterior medial PFC (BA 10) (*β *= 0.36, *t *= 4.25, *p *= 0.0024, *q *= 0.34) and child right TPJ (*β *= 0.36, *t *= 4.19, *p *= 0.0016, *q *= 0.30). As a follow-up test, we conducted an Analysis of Variance comparing the high PA matching condition to the remaining three conditions, which revealed that mother–child neural synchrony was stronger in this condition relative to the other conditions (*F*_43,1450_ = 9.77, *p *< 0.0001).

**Table 2.** Mother–child neural synchrony by affective state matching group

Mother source	Mother detector	Mother region	Child source	Child detector	Child region	*β*	*t*	*p*	*q*
Mother–child matching on high PA									
S9	D14	Right lateral PFC	S4	D6	Right lateral PFC	0.32	3.81	0.0048	0.47
S9	D14	Right lateral PFC	S6	D8	Right TPJ	0.36	4.19	0.0016	0.30
S9	D14	Right lateral PFC	S4	D5	Anterior medial PFC	0.36	4.25	0.0024	0.34
Mother–child mismatch (child high PA, mother low PA)									
S9	D14	Right lateral PFC	S1	D1	Left TPJ	0.39	4.67	0.0024	0.35
Mother–child mismatching (child low PA, mother high PA)									
None	None	None	None	None	None				
Mother–child matching on low PA									
None	None	None	None	None	None				

#### Mother–child mismatch (child high PA, mother low PA)

**Fig. 3.**
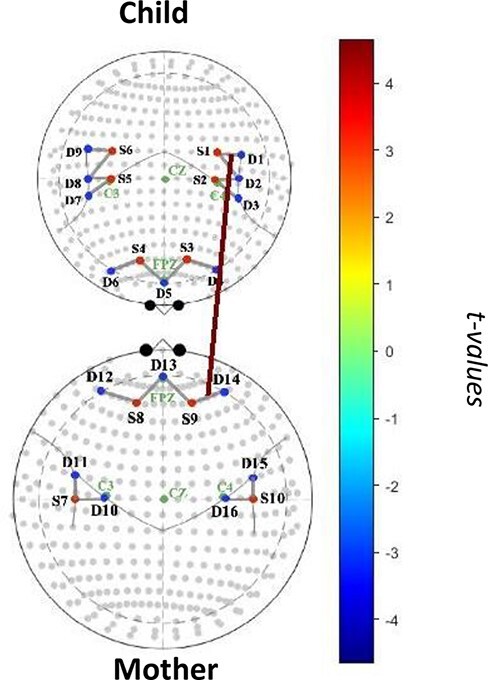
Mother–child neural synchrony during affective mismatch (child high PA, child low PA).

When mothers and children showed a mismatch in PA in which children showed high levels of PA and mothers showed low levels of PA, there was a positive association between mother right lateral PFC activity and child left TPJ activity (*β *= 0.39, *t *= 4.67, *p *= 0.0024, *q *= 0.35, Figure 3).

#### Mother–child mismatch (child low PA, mother high PA)

There was no significant association between mother and child neural activity during moments when children showed low PA and mothers showed high PA.

#### Mother–child matching of low PA

There was no significant association between mother and child neural activity when mothers and children matched on low levels of PA.

### Child age as a moderator of affective and neural synchrony

#### Age as a moderator and mother–child matching of high PA

**Fig. 4.**
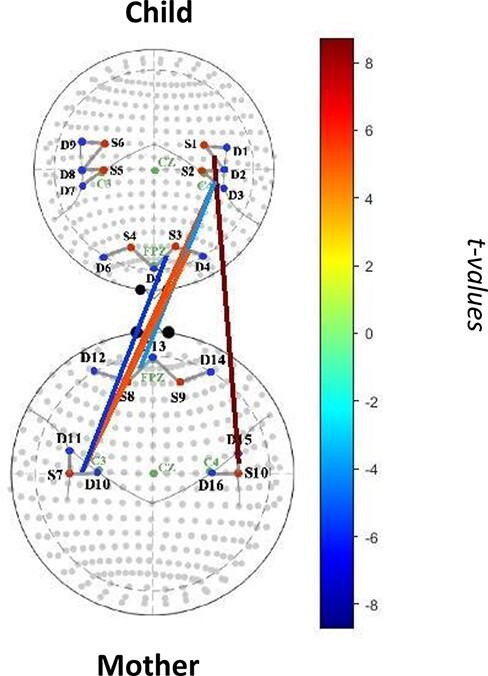
Mother–child neural synchrony during high-intensity positive affective state matching as moderated by child age.

Age moderated the association between mother neural activity and child neural activity such that there was a stronger positive association between mother bilateral TPJ activity and child left TPJ activity for dyads with older children (child left and mother left; *β *= 0.06, *t *= 5.36, *p *= 0.0004, *q *= 0.18; child left and mother right TPJ; *β *= 0.08, *t *= 8.72, *p *= 0.0002, *q *= 0.17). Dyads with older children also showed a stronger positive association between mother activity in the left TPJ and child activity in the left lateral PFC (*β *= 0.05, *t *= 5.20, *p *= 0.0010, *q *= 0.25, see Figure 4).

In contrast, the association between mother anterior medial PFC and child left TPJ activity was stronger when dyads had younger children (*β *= −0.05, *t *= −4.02, *p *= 0.0048, *q *= 0.47). The association between mother left TPJ and child anterior medial PFC activity was stronger for dyads with younger children (*β *= −0.06, *t *= −5.72, *p *= 0.0004, *q *= 0.17).

#### Age as a moderator and other mother–child matching conditions

Age did not moderate the association between mother and child neural activity when children showed high PA but mothers showed low PA, when children showed low PA but mothers showed high PA or when mothers and children matched on low PA (Table 3).

**Table 3.** Age as moderator of mother–child neural synchrony

Mother source	Mother detector	Mother region	Child source	Child detector	Child region	*β*	*t*	*p*	*q*
Mother–child matching on high PA									
S7	D10	Left TPJ	S2	D3	Left TPJ	0.06	5.36	0.0004	0.18
S10	D15	Right TPJ	S1	D2	Left TPJ	0.08	8.72	0.0002	0.18
S7	D10	Left TPJ	S3	D4	Left lateral PFC	0.05	5.20	0.0010	0.25
S7	D10	Left TPJ	S3	D5	Anterior medial PFC	−0.06	−5.72	0.0004	0.17
S8	D13	Anterior medial PFC	S2	D3	Left TPJ	−0.05	−4.02	0.0048	0.47
Mother–child mismatching (child high PA, mother low PA)									
None	None	None	None	None	None				
Mother–child mismatching (child low PA, mother high PA)									
None	None	None	None	None	None				
Mother–child matching on low PA									
None	None	None	None	None	None				

#### Deoxyhemoglobin findings

Findings using deoxyhemoglobin are described in detail in the Supplementary Tables. In short, many of the findings were substantively similar to tests using oxyhemoglobin, especially those effects moderated by age.

## Discussion

Our findings provide evidence that mothers and their young children show synchronized brain activity in neural regions implicated in emotion expression, emotion regulation and mentalizing during periods of interactive play. In particular, our findings illustrate that this neural synchronization seems to be *largely specific* to when mothers and their very young children are matching each other’s high-intensity positive emotional states (relative to matching of neutral or low states). In line with prior behavioral research (Lobo and Lunkenheimer, 2020), this finding may suggest that mother–child co-regulation of neural systems occurs in the context of shared positive emotion and reciprocal and responsive behavior. In addition to prior work demonstrating that mother–child neural synchrony occurs in the context of shared eye gaze, gentle touch and simultaneous vocalizations (Wass *et al*., 2020; Nguyen *et al*., 2021a), our findings suggest that in the first years of life, synchronization of mother–child brain activity may also be occurring during moments in which mothers and children are acknowledging, imitating and elaborating on one another’s positive emotional expressions and behaviors (e.g. sharing smiles, laughs or happy vocalizations) and engaging in joint, back-and-forth play. Still, caution should be taken given that none of our findings passed corrections for multiple comparisons.

Specifically, we found that moments of shared high-intensity positive emotion were associated with mother–child concordant activity in the right lateral PFC, a region implicated in regulation of emotion, including positive emotions (Spielberg *et al*., 2008; Etkin *et al*., 2011). In this regard, during moments of mutual enjoyment, greater activity in this region may allow mothers and children to upregulate their emotions and match each other’s expressions. Indeed, prior empirical and conceptual work has highlighted the need for both dyadic partners’ use of co-regulatory and self-regulatory processes for positive, harmonious dyadic interactions (Bell, 2020).

Furthermore, these moments of shared high-intensity positive emotion were also associated with coordinated activity between the mother’s right lateral PFC and activity in the child’s right TPJ and anterior medial PFC. The right TPJ is involved in understanding the thoughts and feelings of others (Decety and Lamm, 2007), and the anterior medial PFC plays a role in generation of emotion expression (Spielberg *et al*., 2008; Etkin *et al*., 2011). Taken together, child neural activity within the right TPJ may allow children to understand their mothers’ thoughts and feelings, while neural activity in the anterior medial PFC may allow children to express enjoyment and elicit similar reactions from their caregivers. In addition, maternal neural activity within the right lateral PFC may aid mothers in regulating their own emotions to match their child’s affective state. These findings fall in line with a wealth of research demonstrating that caregiver–-child interactions serve to foster child development via mother–child co-regulation of physiological systems such as heart rhythms and hormone release (Feldman *et al*., 2011a,b; Feldman, 2015; Abney *et al*., 2021) and extend this theory to synchronization of neural systems. In this regard, we found that mother–child co-regulation may occur specifically during matching of each other’s positive emotional states.

Intriguingly, during affectively discordant moments in which children showed high levels of PA and mothers showed low levels of PA, we found a positive association between mother right lateral PFC activity and child left TPJ activity. This may suggest that child neural activity in the TPJ may facilitate child understanding of their mother’s lack of positive emotional expression, while greater maternal lateral PFC activity may better allow mothers to upregulate their positive emotions to match their child’s expression (Kim and Hamann, 2007). Of note, there were no significant associations between mother and child brain activity during moments in which mothers showed high PA and children showed low PA.

We also found no significant association between mother and child brain activity when both showed neutral emotion expressions (i.e. low PA). This finding suggests that synchronization of neural systems does not appear to occur when mothers and children are matching one another’s low-intensity emotions but instead occurs only during synchronization of high-intensity positive emotions. Thus, this finding provides specificity that mother–child neural synchronization occurs in the context of mutually positive interactions and falls in line with recent work showing greater mother–infant neural synchrony during times of increased mother–infant proximity and touch (Nguyen *et al*., 2021a).

Our models also evaluated the link between mother–child affective state matching and mother–child neural synchrony using deoxyhemoglobin data. Many of the deoxyhemoglobin findings were substantively similar to findings using oxyhemoglobin (see Supplementary Tables). For example, as with the oxyhemoglobin findings, we found that mother neural activity in the right lateral PFC was associated with child neural activity in the anterior medial PFC during moments of shared positive emotions. We also found similar age effects for our deoxyhemoglobin tests, in that dyads with older children were more likely to have concordant activity in the left TPJ. That our deoxyhemoglobin results mirrored many of our oxyhemoglobin results in our study provides greater validity to our findings.

In line with the vast socioemotional developmental changes occurring from age 1 to 3 (Kopp, 1982), we found that child age moderated the associations between mother and child neural activity during moments of high positive affective matching. Specifically, there was a stronger association between mother bilateral TPJ activity and child left TPJ activity for dyads with older children. This finding falls in line with developmental research demonstrating that mentalizing abilities come online around age 18 months and become increasingly sophisticated thereafter (Frith *et al*., 2003). Furthermore, we found that dyads with older children showed a stronger association between mother activity in the left TPJ and child activity in the left lateral PFC. In this regard, older children may be able to utilize greater activity in the lateral PFC to upregulate their own emotions to match their mothers’ emotion expressions.

In contrast, the association between mother and child anterior medial PFC activity and left TPJ activity was stronger in dyads with younger children. This finding may reflect that in moments in which one member of the dyad is exhibiting high levels of activity in the anterior medial PFC (which facilitates positive emotion expression), the other dyadic member may require greater activity in the left TPJ to understand their partner’s positive emotion expressions perhaps due to aforementioned developmental constraints.

Limitations to our study include varying proportions of affective state groupings (i.e. fewer epochs that featured high PA matching) in our dataset and within dyads, which we accounted for in analyses using nonparametric permutation methods (Fishburn *et al*., 2018). We also note that none of our findings passed formal corrections for multiple comparisons. The use of a task that elicits more frequent and more intense expressions of positive emotion (e.g. bubble-popping task) might have allowed for more instances of mother positive emotion expression, child positive emotion expression and their simultaneous occurrence. Also, a task that elicited other emotions (i.e. frustration, sadness) would have provided an opportunity to evaluate synchronization of brain activity during mother–child matching of negative emotion expression, a process that can escalate negative emotions, thereby promoting emotional dysregulation in children over time (Lobo and Lunkenheimer, 2020). Of note, because we did not assess affective state matching in a manner that allowed for the time dynamics of either up- or down-regulation (see Wass *et al*., 2020; Abney *et al*., 2021), we cannot rule out the possibility that mother–child neural synchronization during periods of high positive affective state matching may simply reflect mutual engagement and enjoyment of one another during a pleasant interactive task rather than co-regulation. Furthermore, our project did not include autonomic measures (e.g. electrocardiography) that could have allowed us to differentiate emotional valence from arousal. Finally, our evaluation of dyads with emotionally and psychiatrically healthy mothers and with children who are developmentally typical also limits the generalizability of our findings to dyads with parental or child psychopathology, child developmental disabilities or other caregiver types (e.g. fathers, grandparents). Future work should utilize tasks that elicit more intense and varied emotional expressions in caregivers and children and should examine how dyadic neural and affective synchrony may be related to child functioning longitudinally.

Nevertheless, our study has multiple strengths including (i) examination of mother–child affective and neural synchrony during a sensitive period of emotional and neural development and (ii) use of cutting-edge technology (i.e. NIRS hyperscanning) that allows for simultaneous assessment of mother–child brain activity during *in vivo* interactions. In sum, our findings provided empirical support that *mutually positive* moments (rather than moments that are mutually neutral or affectively discordant) during mother–child interactions are associated with greater synchronization of brain regions involved in emotion expression, emotion regulation and mentalizing in mothers and their 1- to 3-year-old children. This provides new, clinically important information demonstrating the development of child neural emotional and regulatory regions via caregiver influence. Intriguingly, our findings also point to neural synchronization during moments of affective discordance that may serve to repair the interaction. Overall, our findings support encouraging positive, synchronous play in the first years of life as a means of supporting mother–child co-regulation of child homeostatic systems.

## Supplementary Material

nsad001_Supp
