# Supplementary material for: Mother–child neural synchronization is time linked to mother–child positive affective state matching
Source: Soc Cogn Affect Neurosci. 2023 Jan 27;18(1):nsad001. doi: 10.1093/scan/nsad001 (PMC9976748; doi:10.1093/scan/nsad001)
Supplement: nsad001_Supp [file nsad001_supp.zip › scan-22-060-File002.docx]

| ***Mother-Child Matching on High PA*** | | | | | | | | | | |
| --- | --- | --- | --- | --- | --- | --- | --- | --- | --- | --- |
| Mother Source | Mother Detector | Mother Region | Child Source | Child Detector | Child Region | *β* | *t* | *p* | *q* |  |
| S8 | D13 | Anterior medial PFC | S4 | D5 | Anterior medial PFC | .42 | 4.18 | .0004 | .17 |  |
| S7 | D11 | Left TPJ | S4 | D6 | Right lateral PFC | .29 | 3.26 | .0018 | .30 |  |
| S9 | D14 | Right lateral PFC | S4 | D5 | Anterior medial PFC | .36 | 4.37 | .0036 | .43 |  |
|  | | | | | | | | | |  |
| ***Mother-Child Mismatch (Child High PA, Mother Low PA)*** | | | | | | | | | |  |
| Mother  Source | Mother  Detector | Mother Region | Child Source | Child Detector | Child Region | *β* | *t* | *p* | *q* |  |
| S7 | D11 | Left TPJ | S5 | D7 | Right TPJ | .40 | 4.52 | .0004 | .17 |  |
|  | | | | | | | | | |  |
| ***Mother-Child Mismatch (Child Low PA, Mother High PA)*** | | | | | | | | | |  |
| Mother  Source | Mother  Detector | Mother Region | Child Source | Child Detector | Child Region | *β* | *t* | *p* | *q* |  |
| S8 | D13 | Anterior medial PFC | S4 | D5 | Anterior medial PFC | .44 | 6.55 | .0006 | .18 |  |
|  | | | | | | | | | |  |
| ***Mother-Child Matching of Low PA*** | | | | | | | | | |  |
| Mother  Source | Mother  Detector | Mother Region | Child Source | Child Detector | Child Region | *β* | *t* | *p* | *q* |  |
| None | None | None | None | None | None | -- | -- | -- | -- |  |
|  | | | | | | | | | |  |

**Supplemental Table 1.** Mother-Child Neural Synchrony by Affective State Matching Group: Deoxyhemoglobin findings

| ***Mother-Child Matching on High PA*** | | | | | | | | | | |
| --- | --- | --- | --- | --- | --- | --- | --- | --- | --- | --- |
| Mother Source | Mother Detector | Mother Region | Child Source | Child Detector | Child Region | *β* | *t* | *p* | *q* |  |
| S7 | D10 | Left TPJ | S1 | D1 | Left TPJ | .04 | 3.42 | .0042 | .44 |  |
| S10 | D16 | Right TPJ | S3 | D4 | Left lateral PFC | .05 | 4.98 | .0006 | .18 |  |
| S10 | D15 | Right TPJ | S4 | D5 | Anterior medial PFC | .05 | 4.72 | .0008 | .22 |  |
| S8 | D13 | Anterior medial PFC | S4 | D5 | Anterior medial PFC | -.05 | -3.87 | .0016 | .30 |  |
| S8 | D13 | Anterior medial PFC | S5 | D8 | Right TPJ | -.03 | -3.13 | .0038 | .43 |  |
| S7 | D11 | Left TPJ | S5 | D8 | Right TPJ | -.04 | -2.67 | .0036 | .43 |  |
|  | | | | | | | | | |  |
| ***Mother-Child Mismatch (Child High PA, Mother Low PA)*** | | | | | | | | | |  |
| Mother  Source | Mother  Detector | Mother Region | Child Source | Child Detector | Child Region | *β* | *t* | *p* | *q* |  |
| None | None | None | None | None | None | -- | -- | -- | -- |  |
|  | | | | | | | | | |  |
| ***Mother-Child Mismatch (Child Low PA, Mother High PA)*** | | | | | | | | | |  |
| Mother  Source | Mother  Detector | Mother Region | Child Source | Child Detector | Child Region | *β* | *t* | *p* | *q* |  |
| None | None | None | None | None | None | -- | -- | -- | -- |  |
|  | | | | | | | | | |  |
| ***Mother-Child Matching of Low PA*** | | | | | | | | | |  |
| Mother  Source | Mother  Detector | Mother Region | Child Source | Child Detector | Child Region | *β* | *t* | *p* | *q* |  |
| None | None | None | None | None | None | -- | -- | -- | -- |  |
|  | | | | | | | | | |  |

**Supplemental Table 2.** Age as Moderator of Mother-Child Neural Synchrony: Deoxyhemoglobin Findings
