# Supplementary material for: Mother–child neural synchronization is time linked to mother–child positive affective state matching
Source: Soc Cogn Affect Neurosci. 2023 Jan 27;18(1):nsad001. doi: 10.1093/scan/nsad001 (PMC9976748; doi:10.1093/scan/nsad001)
Supplement: nsad001_Supp [file nsad001_supp.zip › scan-22-060-File003.docx]

Technical Supplement: Mother-Child Synchrony

1. The Sample.

There are 41 mother-child (M,C) dyads. There are up to 36 opportunities for measures per dyad: a 3-min recording period with 5-sec intervals which we call *moments*. We have data for 1411 moments, or 95.6% of the possible 1476 = 41 x 36 because of 7 fussy children.

1. Coding of Affect.

The affect for mother and child at each moment is coded as 1 for high positive affect (PA and 0 for low PA. For dyad *d*, (*MA*(*t|d*)*, CA*(*t|d*)) is the affect pair for mother and child, respectively, at moment *t*. Here, *d* = 1*, . . . ,* 41 and *t* = 1*, . . . ,* 36, with 4.4% of these pairs missing. Thus, for each dyad and each moment the affect pair will be in one of four states: (1,1), (1,0), (0,1), (0,0). The overall proportions for each state are: 6.4%, 15.7%, 9.3%, 68.6%. Thus, 22.1% of mothers’ readings were PA=1, and 15.7% of children’s readings were PA=1.

1. NIRS Pre-Processing

The NIRS probe contained 20 measurement channels (source-detector pairs), 8 for each mother, and 12 for each child; label them (1,. . . ,8,9,. . . 20), the first 8 for mother, and last 12 for child. Each of the 20 channels contains a recorded time course of the light transferred from a light source to a detector position on the head and is recorded at two wavelengths of light (690nm and 830nm). The raw NIRS data (a measure of the intensity of the light at each of the two wavelengths) is first log-normalized by the channel mean to produce a time-course of optical density (OD) changes (OD = -log( I/<I>) ). Optical density at the two wavelengths is then converted to changes in oxy- and deoxy-hemoglobin using the modified Beer-Lambert law with a differential pathlength factor of 6 and a partial-volume correction of 60 for both wavelengths. An “instantaneous correlation” time-course is then computed from all 12 [child] x 8 [mother] channels (48 unique time-courses for both oxy- and deoxy-hemoglobin) by taking the vector length of the two channels, c(i) and c(j), of interest at each time-point ( r(i,j,t) = [c(i,t) * (c(j,t)]/sqrt[<c(i)^2>*<c(j)^2>] ; note that c already has mean zero after the conversion to hemoglobin and the temporal-mean of r (<r(i,j,t)>) is the Pearson’s correlation of c(i) and c(j).

1. First-level Statistical Model.

The time-course of each mother-child channel pair (r(i,j,t)) is then used as the dependent variable (y) of a linear regression model (y = x * β + e). The design matrix x encodes the mother PA, child PA, and their four (2-by-2) joint states, and a DC regressor. An iterative, autoregressively whitened, robust, linear estimator is computed using the AR-IRLS model described in Barker et al. (2013). In brief, this model uses a set of nested iterative algorithms to i) compute an autoregressive noise whitening filter (w) for each signal from the residual of the model and ii) compute a robust statistic weighting filter (s) from a Huber bisquare model of the whitened residual. The two filters are applied to both sides of the model (s*w*y = s*w*x*β + e) to correct for both serially correlated noise (due to systemic physiology) and heavy-tailed noise (due to motion artifacts) in the data. This linear model yields estimates for coefficients of interest (β) and the uncertainty covariance estimate of those coefficient estimates for each mother-child channel and oxy-/deoxy-hemoglobin. There are 384 (48 [channels] * 4 [affect states] * 2 [oxy-/deoxy-hemoglobin]) βs estimated per mother-child pair.

1. Second-level Statistical Model.

We define mother-child synchrony as a positive relation between mother-child affective matching and mother-child NIRS connectivity: for example, a high connectivity metric *r* when the affective state is (1*,* 1). A group-level statistical fixed-effects model was fit (Y = X*Β +E ). The model concatenates all 384 first level βs per mother-child pair over all the dyads (13,824 total entries: 36*384) as the dependent variable (Y). The design matrix is constructed using the condition (the label from the first level NIRS regression with the four affect coded states (1,1), (1,0), (0,1), and (0,0)) and mean-centered child age as independent variables. The model used Wilkinson’s notation “β ~ -1 + condition + condition*age”. This design matrix (X) is constructed from the Kronecker product of the single channel fixed effects model described in the notation above with the 96 (= 48 [channels] * 2 [oxy/deoxy-hemoglobin]) covariates. Since we know the uncertainty covariance from the first-level model for each dyad (Cov B̂(i,j)), this is used to pre-whiten the model (W * Y = W * X * Β + E; where W is the whitening filter matrix constructed from the block-diagonal concatenation of the inverse of the Cholesky-decomposition matrix of all the Cov B̂(i,j)’s). Thus, the noise for each dyad is accounted for based on the known first-level model rather than including dyad as a random effect in the model. This model is then solved using an iterative robust statistical estimator similar to the first-level regression where the weighting matrix (S) is defined from the Huber bisquare model of the residual (E). The final model is then S*W*Y = S*W*Β + E.

6. Inference

We used a permutation test for non-parametric statistical inference of the second-level model. For each child, the data from a randomly assigned “mother” was paired as a null-dyad and run through the entire analysis pipeline for first- and second-level models. For the null dyad, the proper age of the child and their proper affect encoding vector was used, but they were paired with a random non-parent’s data. There are 41! *~* 3*.*34 *×* 10^49^ different ways of assigning children to mothers. We model the null hypothesis of no relation between mother and child by choosing at random 20,000 dyadic permutations, computed the synchrony measure for each; the non-parametric *p*-value estimate is the proportion of permutations that yield a more extreme synchrony measure than what we observed with the actual dyads in item 5. The null distribution of each channel-condition was considered separately. Finally, there are many tests, so we used the Benjamini-Hochberg method to assess the false discovery rate with alpha = 0.05.

1
